# Pickleball Injuries in the Aging Athlete: A Critical Analysis Review

**DOI:** 10.7759/cureus.69950

**Published:** 2024-09-22

**Authors:** Daniel C Touhey, Christopher K Bozorgmehr, Darius S Tartibi, Matthew V Smith, Derrick M Knapik

**Affiliations:** 1 Orthopaedic Surgery, Washington University Orthopaedics, St. Louis, USA

**Keywords:** aging athlete, athletic injury, management, musculoskeletal, pickleball

## Abstract

The popularity of pickleball has rapidly increased in the United States, with over 48 million adult participants in recent years, leading to a growing need to better understand injuries exclusive to pickleball. Pickleball-related injuries are predominantly musculoskeletal, most commonly sprains and strains to the upper and lower extremities. Sport-specific variables, including sporting equipment, playing conditions, and proper technique may be utilized to guide clinicians in injury management and preventative treatment. Future research is warranted to better direct care for pickleball athletes with a focus on the aging population.

## Introduction and background

While initially introduced in 1965 [1], pickleball has become one of the fastest-growing sports in the United States. Pickleball is a paddle sport that draws inspiration from badminton and tennis, where a perforated ball is hit back and forth over a 36-inch net [1]. According to the Association of Pickleball Professionals, over 48.3 million adults played on at least one occasion in 2022, accounting for 19% of the total US population [2]. Since 2017, the number of pickleball players has grown by 85.7% [3], especially among older individuals [2].

As a result of the growing popularity and participation in pickleball, there has been growing interest in better understanding the various injury risks associated with participation. This has led to an increase in the number of published investigations examining injury types, mechanisms of injury, as well as methods to minimize injury incidence [4-7]. Recently, Pergolizzi et al. categorized and described pickleball-related injuries, outlining differences in injury acuity, mechanisms, and patterns based on patient sex [8]. However, no recent investigation has summarized reported injury incidence and etiology, especially in older patients. As such, the purpose of this review is to provide a concise overview of the current literature examining injury types, mechanisms, and current recommendations to decrease injury incidence and severity in patients participating in pickleball.

## Review

Injury epidemiology

As the reported prevalence of pickleball-related injuries has increased, a growing diversity of associated injuries has been documented (Table 1). The predominant type of injury occurring during pickleball is musculoskeletal [4-7]. Two studies reported data on pickleball-related injuries from the National Electronic Injury Surveillance System (NEISS), a database reporting emergency room visits from approximately 100 hospitals across the United States [4,7]. Specifically, Forrester reported in his 2020 study that injuries were most commonly sustained in the lower extremities, consisting of strain/sprain, fracture, and contusions/abrasions [4]. Meanwhile, Weiss et al. reported that the upper extremities were most commonly affected, with strain/sprain, fracture, and contusions/abrasions being the most common [7]. This difference is likely attributable to methodological differences and the date of analysis. Weiss et al. utilized a wider range of search criteria to capture pickleball-related injuries reported through 2019, during which time the sport had increased in popularity; while Forrester’s search extended through 2017, resulting in fewer reported injuries [4,7].

**Table 1:** Overview of Included Studies AT = Achilles tendon; HT = Hamstring tendon; Med = Medial; Lat = Lateral; LE = Lower extremity; PF = Plantar fascia; PT = Peroneal tendon; PVD = Posterior vitreous detachment; UE = Upper extremity *Numerical values represent estimates of injury incidence from 2013-2022, based on weighted calculations from the NEISS (National Electronic Injury Surveillance System) †Includes 33 paddleball-related injuries ‡Includes 38 paddleball-related injuries

Study	No. of patients	% of Males	Mean age (years)	Body region injured	Injury diagnosis
Atkinson et al.(2022) [9]	2	50%	63	Eye (n=2)	PVD + retinal tear + retinal detachment (n=1); PVD + retinal tear (n=1)
Firouzbakht et al.(2024) [10]	12,021	30%	65.5	Hand/wrist (n=12,021)*	Fracture (n=7,153), strain/sprain (n=2,120), contusion/abrasion (n=755), dislocation (n=739), pain/tingling (n=710), laceration (n=544)
Forrester (2020) [4]	300	50%	63	LE (n=95), UE (n=79), trunk (n=64), head/neck (n=50), all of body (n=12)	Strain/sprain (n=87), fracture (n=87), contusion/abrasion (n=33), laceration (n=15), internal injury (n=18), dislocation (n=8), concussion (n=3), hematoma (n=1), avulsion (n=1); other (n=47)
Gervais and Diggle (2023) [11]	1	0%	67	Hand/wrist (n=1)	Distal radial cutaneous sensory neuropathy (n=1)
Huang and Greven (2024) [12]	2	50%	76.5	Eye (n=2)	Intraocular lens subluxation and capsular bag complex displacement (n=1); Traumatic iritis + iris sphincter tear + crystalline lens subluxation (n=1)
Kasper et al. (2023) [5]	171	33%	65	Upper extremity (n=204)†	Wrist fracture (n=64), unknown (n=62), lat/med epicondylitis (n=30), metacarpal fracture (n=17), tendonitis (n=10), other (n=10), tenosynovitis (n=7), trigger finger (n=4)
Opara et al. (2024) [6]	128	56%	63	Lower extremity (n=166)‡	Ankle sprain/strain (n=36), AT rupture (n=20), AT tendonitis (n=14), AT bursitis (n=10), LE fractures (n=18), LE contusions (n=3), PT tendonitis (n=5), PT rupture (n=1), flexor tendon rupture (n=12), plantar fasciitis (n=11), PF rupture (n=6), PF fibromatosis (n=6), other (n=24)
Weiss et al. (2021) [7]	523	51%	66	UE (n=172), LE (n=156), head/neck (n=103), trunk (n=82), all of body (n=10)	Strain/sprain (n=170), fracture (n=150), contusions/abrasions (n=55), internal injury (n=51), other (n=47), laceration (n=27), dislocation (n=15), concussion (n=3), hematoma (n=5)
Vitale and Liu (2020) [13]	1	100%	71	Hamstrings/thigh (n=1)	Full-thickness HT avulsion + full-thickness adductor magnus tendon rupture (n=1)

Two separate case series have reported ocular trauma in four pickleball athletes, with two cases necessitating surgical treatment [9,12]. Additionally, a recent case study documented the onset of pickleball-induced peripheral neuropathy in a 67-year-old man [11]. Although current studies are limited due to small sample sizes, these cases demonstrate the niche potential of pickleball to cause major injury in an older demographic that is highly representative of the current population of athletes.

Given the demands of the sport and interest in the older population, multiple investigations have focused on the prevalence of injuries in older patients [4-7]. The elderly population is at a uniquely higher risk of musculoskeletal injury due to various anatomic and physiologic factors. It is well documented that age-related changes in the collagen composition of tendons and ligaments lead to decreased tissue compliance and increased susceptibility to injury [14,15]. Likewise, decreases in skeletal muscle mass lead to reduced force production, less flexibility, and lower overall physical function [16]. As muscles fatigue and weaken through extended periods of activity, flexibility decreases while absorbing energy during eccentric muscle activities [14], such as planting or stopping motions performed in pickleball, placing participants at greater risk for injury. Together, these changes increase the propensity for acute injuries such as muscle, tendon, and ligament strains and sprains, which make up the most frequently reported acute injuries among aging athletes [4,6,7,14,16]. Specifically, among musculoskeletal injuries sustained in pickleball, strains and sprains are most common, with Forrester reporting strains/sprains occurring in 28.7% of injuries, while Weiss et al. reported a 33.2% prevalence [4,7]. Weiss et al. additionally reported that men were 3.5 times more likely to sustain a strain or sprain than females with the lower leg (usually the calf or Achilles tendon) most commonly affected [7]. Opara et al. reported on lower body injuries in pickleball, reporting that ankle sprains/strains from a twisting mechanism were the most frequently reported, representing 21.7% of reported injuries [6]. Vitale and Liu reported a single case of a 71-year-old male pickleball athlete who sustained an avulsion fracture with a full-thickness tear of the proximal hamstring, along with a full-thickness tear of the adductor magnus [13]. Although the patient recalled acute symptoms while lunging for a ball, antecedent symptoms indicated an acute-on-chronic injury [13].

Similarly, the progressive annual decrease in bone mineral density after age 40, ranging from 0.5 to 0.75% in men and 1.5 to 2% in women, places older athletes at a greater risk for fracture [16]. Following strains and sprains, upper and lower extremity fractures are the next most commonly reported injuries among aging athletes [4,6,7,16-18]. Fractures were the second most commonly reported injury as reported by Forrester (27.7%) and Weiss et al. (28.1%) [4,7]. Weiss et al. additionally reported that women were over 3.5 times more likely to sustain a fracture, and nine times more likely to sustain a wrist fracture during pickleball when compared to males [7]. Bony fractures are the most frequently reported upper extremity injuries among aging pickleball athletes as reported by Firouzbakht et al. at 60.3% of total estimated pickleball injuries in the US between 2013 and 2022 [10]. Kasper et al. likewise reported that fractures were the most common injury at 39.7% of total reported upper extremity injuries in a single orthopaedic practice between 2015 and 2022 [5]. More specifically, wrist fractures sustained during slips/falls/dives were the most common fracture type among pickleball athletes at 50.0% and 31.4% of total estimated and reported injuries by Firouzbakht et al. and Kasper et al., respectively [5,10]. Kasper further reported that pickleball athletes over 65 years of age who sustain a wrist fracture were more likely to undergo surgery when compared to the general population [5].

Analysis of acute and chronic injuries occurring during racquet sports other than pickleball reveal common injury patterns among recreational and competitive-level athletes. Several studies have cited acute lower extremity injuries as the most prevalent injury type among tennis players, with ankle injuries being the most common [19-22]. Specifically, inversion ankle sprains affecting the lateral ligaments (i.e., anterior talofibular ligament, calcaneofibular ligament, and posterior talofibular ligaments) predominate [23]. Similar to the analysis of pickleball injuries by Opara et al., tennis-related ankle injuries occur from twisting forces produced during the frequent running and pivoting, stopping and starting movements, such as lunging and jumping, that are required during competition [22]. Following ankle injuries, acute injuries to the wrist, knee, foot, and shoulder are next most common among elite-level tennis players [19]. Muscle and tendon injuries likewise predominate among tennis athletes as with pickleball players who sustain strains and sprains above any other injury type [19,20,23]. Beyond tennis, squash players are reported to experience proportionally more acute rather than chronic injuries, with the lower extremities being most commonly affected [24,25]. Similar to pickleball, badminton injuries are reported to involve the lower extremities most commonly [25,26], while acute sprains to the lower extremities and upper extremity tendinopathies are reported to be most common among racquetball athletes [24,25].

Musculoskeletal injuries sustained during pickleball participation are predominantly managed non-operatively. Kasper et al. and Opara et al. reported that a combined 86% (n=480/561) of all treatments for upper and lower extremity pickleball-related injuries at a single orthopaedic practice involved non-operative modalities. These treatments included bracing (54%, n=261/480), physical therapy (34%, n=165/480), and steroid injections (11%, n=54/480) [5,6]. In this same population of pickleball athletes reported by Kasper et al. and Opara et al., 14% (n=81/561) of cases were surgical, most commonly consisting of Achilles tendon repair (46%, n=37/81), open reduction and internal fixation (ORIF) of a wrist fracture (35%, n=28/81), and distal bicep tendon repair (4%, n=3/81) [5,6]. Additional surgical treatments have been reported for pickleball-related ocular injuries, including two cases of scleral-fixated intraocular lens implantation following traumatic lens subluxation [12].

Despite the predominance of studies evaluating injuries in elderly athletes, recent literature has examined injuries in younger pickleball players [2]. In a prospective cohort study of 73 elite junior-level tennis players (ages 11-14 years) throughout a single season (32 weeks) [19,27], overuse injuries comprised the greatest proportion of total reported health problems over the study period (47%, n=88/187), with acute injuries reported less frequently (13%, n=25/187). Additional studies have reported back injuries to be the most common among junior tennis athletes (ages 10-18 years), followed by injuries to the shoulder, ankle, and wrist [19,22,28-30]. Nonetheless, in a series of 600 competitive league tennis athletes with a mean age of 24.8 years [20], acute injuries affecting the lower extremities, specifically lateral ankle injuries, were reported to be the most prevalent. This data indicates that injury trends in racquet sports become more similar to those reported in pickleball with the increasing age of the athletes. Given the scarcity of data surrounding injuries in junior-level pickleball, future research should seek to stratify pickleball-related injuries by demographic to critically evaluate how injuries differ across various age groups as the sport becomes more widely played.

Sport-specific factors contributing to injury

While pickleball draws inspiration from other net sports, certain injuries and risk factors unique to participation in pickleball have been identified. In their systematic review, Prayudo et al. identify eight risk factors for overall pickleball-related injuries listed in the literature, including advanced age, poor technique and equipment, improper stretching/warm-up, past injury, and court surface [31]. While these risk factors may predict injury in a variety of sports, advanced age is important in the context of the sports increasing popularity among older athletes. In other racquet sports, the average injury age is reported to be under 50 years [32], while in pickleball, a high number of reported cases are reported in patients aged 60 years and older [4-7]. Considerations for elderly athletes suggest that reductions in reaction time may lead to more acute, traumatic injuries [33], in addition to increases in slips and falls.

Additional sport-specific factors leading to injury during pickleball participation include equipment considerations. Specifically, players new to the game tend to use heavier paddles, for example, those weighing 8.5 oz, compared to lighter versions that weigh closer to 7.2 oz [13]. This small difference may place beginners at an increased risk of lateral epicondylitis. Novice players are advised to start with a continental, or neutral grip, which evenly supports forehand and backhand strokes, and to grip the paddle loosely to avoid elbow pain [13]. The equally popular Eastern grip, which slightly favors the forehand, has been related to radial-sided injuries (e.g., flexor carpi radialis tendinitis) while the Western grip, which more heavily favors the forehand, is associated with ulnar-sided injury (e.g., extensor carpi ulnaris tendinitis) [23,34]. Pickleballs of varying weights may be used depending on the play setting, with lighter, slower balls appropriate for indoor play and heavier, faster balls more appropriate for outdoor conditions. These subtle weight changes may increase injury risk in less experienced players, as faster conditions correspond to a greater proportion of musculoskeletal injury and ball-contact injuries to the head and face [10,13,23].

Court-related considerations also play an important factor in injury risk during pickleball participation. While smaller than a tennis court (average court size 20 x 44 ft), pickleball courts may increase the likelihood of player-player contact injury during doubles competition [12]. Depending on the setting, outdoor courts may also contain wet surfaces, cracks, or bare spots, which may be difficult to detect by older athletes, leading to falls [10,13]. Furthermore, different surface types present various advantages and drawbacks [35]. For instance, acrylic and rubber offer a good grip for planting and cutting, while concrete and polypropylene become slippery with the accumulation of moisture on their surface. Wood courts likewise offer improved traction but may become slick when wet. Concrete surfaces are more impactful on joints, while rubber and synthetic turf offer improved wear properties over the longevity of play. Proper footwear is another consideration; sneakers designed for court play should offer sufficient traction and multidirectional support, in contrast to running shoes designed for unidirectional motion [13]. Improper footwear may lead to a higher instance of foot and ankle injury, especially ankle sprains.

Precautions

Currently, there is limited evidence-based sport-specific guidance on methods to decrease the risk of injuries in athletes participating in pickleball. In addition to reporting descriptive injury data, few suggestions aimed at decreasing injury incidence have been reported. As a pickleball point develops, players often race to the front of their court to the “non-volley zone” adjacent to the net, requiring the need to make sudden stops to maintain volley eligibility. Vitale and Liu recommend that patients take precautions when making these eccentric stops to reduce lower extremity musculoskeletal burden [13]. More generally, proper equipment is advised. Protective glasses can prevent acute, traumatic, ocular injury [9,12,13]. Additionally, well-fitting shoes are recommended to prevent acute and chronic musculoskeletal injury, while an appropriately weighted racquet can reduce overuse injuries in the upper extremities [13].

Additional suggestions involve exercise and fitness regimens to aid in preventing injury, especially in older athletes. To avoid muscular injury, care should be taken to appropriately warm up before beginning competitive pickleball play. Flexibility exercises are strongly recommended to optimize performance, maintain range of motion, and decrease the risk of injury among aging athletes [16]. Multiphase training, including muscle strengthening and assessments of balance, coordination, and reaction time, should be part of a fitness routine [36]. Resistance training has proven to increase muscle strength, bone mass, and bone mineral density (BMD) in active aging individuals, particularly in female athletes who begin losing BMD at a younger age and faster rate when compared to males [16]. Furthermore, aging athletes consistently exhibit higher indicators of bone health than their less athletic counterparts, coinciding with greater fracture resistance. Specifically, Korhonen et al. demonstrated that bone parameters (e.g., tibial midshaft bending strength, distal and midshaft tibial bone mineral content) are 11-48% higher in competitive Masters-level sprinters (ages 40 to 85 years) compared to healthy, active controls (aged 31 to 45 years) [16,37].

## Conclusions

As pickleball popularity and accessibility continue to increase, we present a narrative review of the reported injury data. Although there have been cases of traumatic ocular injury and neuropathic pain, reported pickleball injuries are predominantly musculoskeletal. Cases tend to be in older athletes with pattern differences based on the body region impacted. Upper extremity pickleball injuries are more likely to result from falls, while lower extremity injuries are more often attributed to non-fall movements. However, the reported data suggests that elderly participants are at slightly increased risk for injury and that adequate equipment, preparation, and in-game technique may reduce the risk of chronic and acute injuries.
